# Electro-Optical Correlation of Charge-Transfer Kinetics in Composition-Tuned Wurtzite Oxide Anodes for Sodium-Ion Storage

**DOI:** 10.3390/ma19173643

**Published:** 2026-08-27

**Authors:** Abrar Hussain, Muneer Hussain, Muhammad Tahir Khan, Dabin Park, Haleem Ud Din, Nojoon Myoung, Sisi Wang, Yoonkook Son

**Affiliations:** 1 Department of Basic Sciences, Riphah International University, Islamabad 45320, Pakistan; abrarhussain2014@hotmail.com (A.H.); hussainmuneer61@chosun.ac.kr (M.H.); mtahir.khan@riphah.edu.pk (M.T.K.); 2 Nanoscience and Technology Department, National Centre for Physics, Islamabad 45320, Pakistan; 3 Department of Electrical Engineering, Chosun University, Dong-gu, Gwangju 61452, Republic of Korea; c20217309@gmail.com; 4 Department of Physics Education, Chosun University, Dong-gu, Gwangju 61452, Republic of Korea; haleem.uddin@yahoo.com (H.U.D.); nmyoung@chosun.ac.kr (N.M.); 5 Institute of Well-Aging Medicare & Chosun University G-LAMP Project Group, Chosun University, Dong-gu, Gwangju 61452, Republic of Korea; 6 Key Laboratory of Urban Rail Transit Intelligent Operation and Maintenance Technology & Equipment of Zhejiang Province, College of Engineering, Zhejiang Normal University, Jinhua 321004, China

**Keywords:** sodium-ion batteries, anode, DFT, ZnO, conversion/alloying reaction

## Abstract

Composition-tuned Zn_1−x_Cu_x_O (x = 0.0, 0.1, and 0.2) nanoparticles were synthesized via a facile co-precipitation method and investigated as wurtzite oxide anodes for sodium-ion batteries (SIBs). This work focuses on establishing an electro-optical correlation between optical band-edge modulation and electrochemical charge-transfer kinetics. X-ray diffraction confirmed the formation of the hexagonal wurtzite ZnO phase without detectable secondary phases, while FESEM and particle-size distribution analysis showed that the Zn_0.9_Cu_0.1_O sample possessed a more refined and uniform particle population than pristine ZnO. Diffuse reflectance spectroscopy (DRS) revealed a non-linear optical response with composition, where the direct optical band gap decreased from 3.08 eV for pristine ZnO to 2.66 eV for Zn_0.9_Cu_0.1_O, followed by partial recovery to 2.99 eV for Zn_0.8_Cu_0.2_O. Density functional theory (DFT) calculations supported this trend by showing the appearance of additional electronic states near the Fermi level after Cu incorporation, indicating improved electronic accessibility. Electrochemical impedance spectroscopy further confirmed that Zn_0.9_Cu_0.1_O exhibited the lowest specific charge-transfer resistance of 185.15 Ω cm^2^ and the highest Na^+^ diffusion coefficient of 5.77 × 10^−11^ cm^2^ s^−1^, compared with pristine ZnO and Zn_0.8_Cu_0.2_O. The improved sodium storage performance of Zn_0.9_Cu_0.1_O is, therefore, attributed to the favorable combination of particle uniformity, band-edge modulation, and interfacial charge-transfer kinetics. These findings demonstrate a composition-dependent association between optical band gap modulation and charge-transfer behavior in semiconductor-type oxide anodes for sodium-ion storage.

## 1. Introduction

Sodium-ion batteries (SIBs) are being widely explored as low-cost energy storage systems, especially for large-scale and stationary applications. Their interest mainly comes from the high natural abundance and wide distribution of sodium resources, which can reduce concerns related to raw material cost and supply chain instability [1,2,3]. Lithium-ion batteries (LIBs) are still the dominant technology for portable electronics and electric vehicles because they provide high energy density and are supported by well-developed manufacturing routes. However, the continued expansion of LIBs depends on critical resources such as lithium, cobalt, nickel, and battery-grade graphite, whose availability and price can be affected by geographical concentration and market fluctuation [4,5]. In contrast, sodium is naturally abundant, geographically widespread, and chemically similar to lithium, making SIBs attractive for stationary storage and low-cost energy applications [6]. However, Na^+^ storage is generally more challenging than Li^+^ storage because Na^+^ has a relatively large ionic radius of about 1.02 Å and a higher atomic mass of 22.989 g mol^−1^. The larger ionic size of Na^+^ can hinder ion transport within electrode materials and aggravate structural changes during repeated sodiation/desodiation [7]. These transport and structural limitations can consequently contribute to slower reaction kinetics and reduced electrochemical reversibility [8]. Therefore, the development of suitable electrode materials, especially anode materials with fast charge-transfer behavior and stable sodium storage performance, remains a key challenge for practical SIBs [6,9].

Compared with carbon-based anodes, transition-metal oxides (TMOs) and related metal oxides have received significant attention because they can deliver higher theoretical capacities through conversion, alloying, or combined conversion/alloying reactions. These materials possess several advantages, including low cost, compositional tunability, multiple redox-active centers, and the possibility of tailoring their crystal structure, morphology, and electronic properties [10]. However, TMO anodes generally suffer from poor intrinsic electronic conductivity, large volume expansion during discharge/charge, slow Na^+^ diffusion, and unstable electrode/electrolyte interfaces [11,12]. These drawbacks often result in high polarization, poor rate performance, and rapid capacity decay. Therefore, improving both the structural stability and charge-transfer kinetics of oxide anodes is essential for realizing high-performance SIBs.

Among different oxide anodes, zinc oxide (ZnO) is an interesting wurtzite-structured semiconductor because of its low cost, environmental compatibility, simple crystal structure, good chemical stability, and wide use in optoelectronic, photocatalytic, sensing, and electrochemical systems [13,14]. ZnO has also been investigated as an anode material for lithium-ion and sodium-ion batteries because Zn can participate in reversible alloying reactions after the initial conversion of ZnO [15]. In sodium-ion storage, ZnO is generally considered to undergo conversion to metallic Zn and Na_2_O, followed by Na-Zn alloying/dealloying during subsequent charge/discharge processes [16]. This combined reaction mechanism can provide high capacity, but the practical performance of pristine ZnO is commonly restricted by low electronic conductivity, particle aggregation, poor reaction uniformity, and structural stress generated during repeated sodiation/desodiation.

To address the poor conductivity and structural instability of ZnO-based anodes, previous studies have introduced conductive networks, hybrid architectures, and interface-modified structures [17]. For example, Wang et al. developed a ZnO/rGO/C composite from a metal–organic framework (MOF) precursor, in which the carbon-containing framework enhanced electron transport and helped buffer structural changes during cycling [18]. Jing et al. combined ZnO nanorods with rGO for sodium-ion storage, showing that the graphene network improved both cycling retention and rate response [15]. Feng et al. recently constructed a nitrogen-doped C-coated ZnO/ZnS heterostructure, where the improved electrochemical behavior was associated with enhanced conductivity, internal electric-field effects, and better structural accommodation during sodium storage [8]. Similarly, Teng et al. developed amorphous carbon-coated porous ZnO nanosheets for sodium-ion storage, where the conductive carbon coating and porous architecture improved the electrochemical utilization and cycling behavior of ZnO [19]. These studies confirm that ZnO-based materials are useful platforms for sodium storage research; however, most reported works have mainly focused on carbon coating, heterostructure construction, morphology control, or interface stabilization.

In addition to morphology and interface engineering, elemental doping provides another route for tuning the intrinsic structural and electronic properties of ZnO. In particular, Cu incorporation into ZnO has been widely investigated because it can modify the lattice environment, defect states, optical absorption, band-edge structure, and electrical properties of ZnO [20,21]. Previous studies on Cu-doped ZnO have, therefore, established composition-dependent modification of its optical/electronic characteristics. However, these studies have mainly focused on the fundamental optoelectronic properties of Cu-doped ZnO rather than its sodium storage behavior. Doping has also been explored directly for ZnO-based sodium-ion battery anodes. Hussain et al. investigated Mn-doped ZnO nanoparticles and demonstrated that transition metal incorporation can significantly improve the sodium storage performance of ZnO [22].

Related studies on other semiconductor-type electrodes have also considered relationships between optical/electronic properties and electrochemical behavior. For example, Zhao et al. reported that surface-engineered TiO_2_ anodes for SIBs exhibited simultaneous band gap modification and improved ion/electron transport [23]. Similarly, Iffer et al. combined optical analysis, DFT calculations, and EIS measurements to investigate semiconductor electronic characteristics and electrochemical kinetics in NaVOPO_4_ [24]. Nevertheless, to the best of our knowledge, the composition-dependent correlation among DRS-derived band-edge modulation, DFT-predicted electronic-state evolution, and EIS-derived charge-transfer kinetics has not been jointly investigated for Cu-substituted ZnO anodes in sodium-ion batteries.

In this work, composition-tuned Zn_1−x_Cu_x_O nanoparticles (x = 0.0, 0.1, and 0.2) were synthesized via a co-precipitation method and investigated as anode electrodes for SIBs. The main objective of this study was to establish an electro-optical correlation between DRS-derived band-edge modulation, DFT-predicted electronic-state evolution, and EIS-derived charge-transfer kinetics. DRS analysis revealed a clear non-linear variation in the optical band gap with composition. The direct optical band gap decreased from 3.08 eV for pristine ZnO to 2.66 eV for Zn_0.9_Cu_0.1_O, indicating strong band-edge modification and improved electronic accessibility. However, when the composition was further increased to Zn_0.8_Cu_0.2_O, the band gap partially recovered to 2.99 eV, showing that the strongest band-edge modification among the investigated compositions occurred at x = 0.1 rather than increasing continuously with Cu content. DFT calculations supported this optical trend by showing the formation of additional electronic states near the Fermi level after Cu incorporation, which can facilitate electron transport during the sodium storage reaction. A similar composition-dependent trend was observed in the electrochemical impedance results, where Zn_0.9_Cu_0.1_O exhibited the most favorable charge-transfer behavior among the investigated electrodes.

## 2. Experimental Section

### 2.1. Sample Preparation

**Figure 1:**
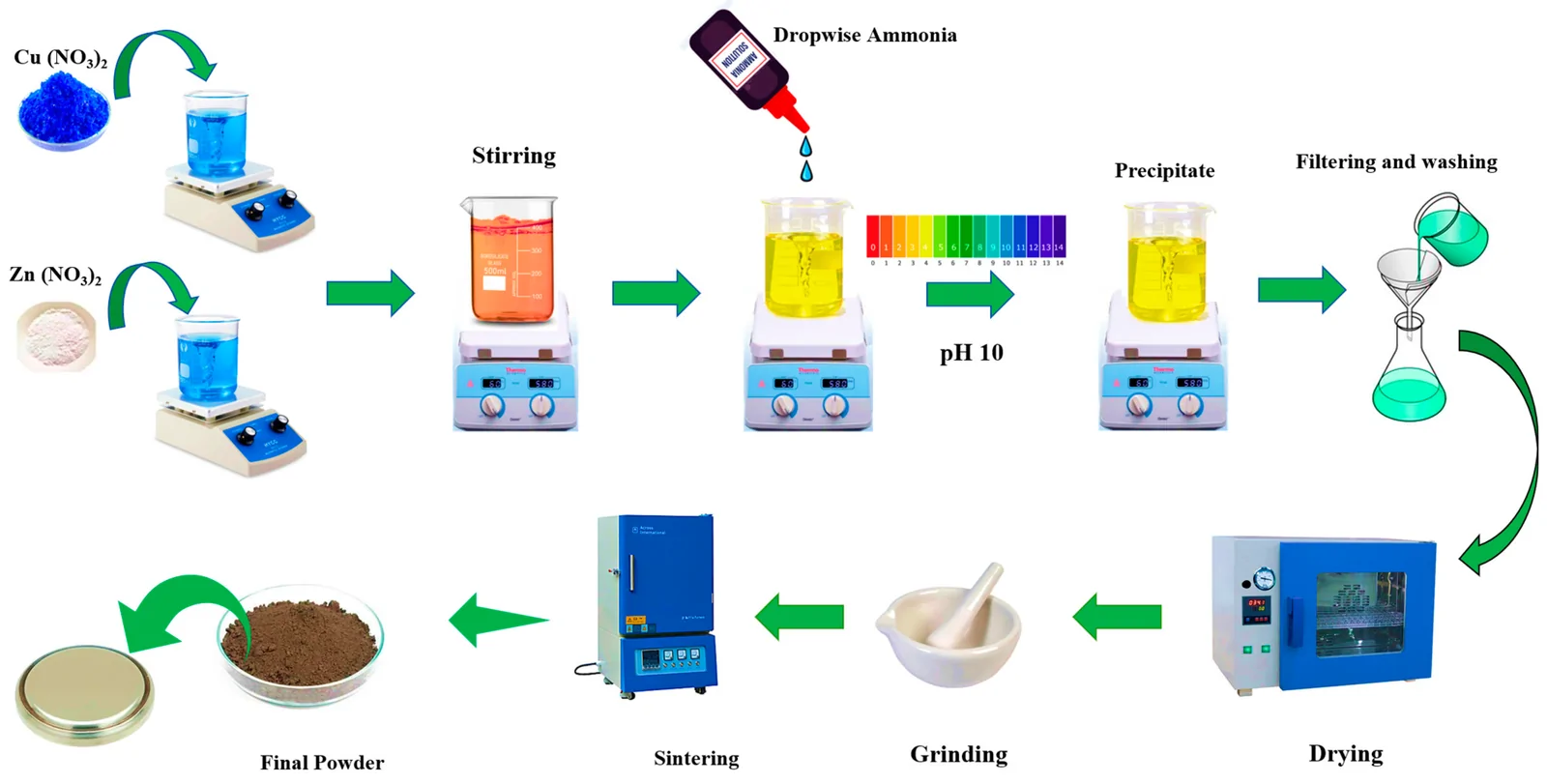
Schematic representation of the co-precipitation route used to synthesize Zn_1−x_Cu_x_O nanoparticles with x = 0.0, 0.1, and 0.2.

Zn_1−x_Cu_x_O nanopowders with nominal compositions of x = 0.0, 0.1, and 0.2 were prepared by a co-precipitation method, as shown schematically in Figure 1. For the undoped ZnO sample, zinc nitrate hexahydrate [Zn (NO_3_)_2_·6H_2_O] (Daejung Chemicals & Metals Co., Ltd., Siheung-si, Gyeonggi-do, Republic of Korea) was used as the Zn source. The required precursor was added to deionized water and magnetically stirred to form a clear zinc-containing solution. During dissolution, the solution temperature was maintained at 30 °C to support uniform mixing. After the precursor was completely dissolved, NH_4_OH solution (35%) (Samchun Pure Chemical Co., Ltd., Pyeongtaek-si, Gyeonggi-do, Republic of Korea) was supplied slowly to the reaction mixture while stirring was continued. The addition of ammonia increased the solution pH to about 10 and immediately produced a white precipitate. This precipitate was associated with the formation of zinc hydroxide intermediate species, which were later converted into ZnO after thermal treatment.

After precipitation, the reaction mixture was kept under continuous stirring for another 1 h to allow the hydroxide precursor to develop uniformly. The solid phase was then separated from the mother solution by filtration through Whatman filter paper. To remove adsorbed impurities, excess ammonia, nitrate species, and other soluble residues, the collected precipitate was washed several times with deionized water and ethanol. The cleaned precipitate was dried at 120 °C for 12 h until the remaining moisture and solvent were removed. The dried solid was then crushed and homogenized using an agate mortar (DAIHAN Scientific Co., Ltd., Wonju-si, Gangwon-do, Republic of Korea). Finally, the obtained powder was heat-treated at 500 °C for 2 h in a muffle furnace, which converted the hydroxide precursor into crystalline ZnO. The calcined powder was stored in a sealed container and used for subsequent structural, morphological, optical, and electrochemical investigations.

For Cu-containing samples, Zn_1−x_Cu_x_O compositions with x = 0.1 and 0.2 were prepared by adjusting the molar ratio of Zn and Cu precursors. Zinc nitrate hexahydrate [Zn (NO_3_)_2_·6H_2_O] and copper nitrate trihydrate [Cu (NO_3_)_2_·3H_2_O] were used as the metal sources. Each salt was dissolved separately in deionized water at 30 °C under magnetic stirring to obtain clear precursor solutions. The two solutions were then combined and stirred further to ensure uniform mixing of Zn^2+^ and Cu^2+^ ions before precipitation. After complete mixing, NH_4_OH solution was slowly added to the mixed metal-ion solution while stirring was continued. The pH was controlled at approximately 10, resulting in the formation of a hydroxide-based precipitate containing both Zn and Cu species. The suspension was maintained under stirring for another 1 h to support complete precipitation and improve compositional uniformity. The solid product was then isolated by filtration, followed by repeated washing with deionized water and ethanol to remove residual ions, nitrates, and unreacted species. The washed precipitates were dried at 120 °C for 12 h and then ground using an agate mortar to obtain fine powders. Finally, the dried precursors were calcined at 500 °C for 2 h in air to form crystalline Zn_0.9_Cu_0.1_O and Zn_0.8_Cu_0.2_O nanopowders. The obtained materials were stored in sealed vials before further characterization and electrode fabrication.

### 2.2. Material Characterization

The prepared Zn_1−x_Cu_x_O nanopowders were examined through structural, microstructural, compositional, optical, and electrochemical analyses. The XRD patterns were recorded on a Bruker D8 Advance diffractometer (Bruker AXS GmbH, Karlsruhe, Germany) using Cu Kα radiation with a wavelength of 1.5406 Å. FESEM images were collected with a Sigma 500VP instrument (Carl Zeiss Microscopy GmbH, Oberkochen, Germany) to observe the surface features and particle arrangement of the synthesized powders. The same microscopy platform was equipped with an EDX detector, which was used to examine the elemental profile of the Zn_0.9_Cu_0.1_O sample. The optical response of the prepared materials was studied by DRS using a PerkinElmer Lambda-950 UV–Vis–NIR spectrophotometer (PerkinElmer, Waltham, MA, USA). The sodium storage behavior of the electrodes was examined using CV, GCD, cycling stability, rate capability tests, and EIS. For electrochemical measurements, including CV, GCD, cycling stability, rate capability, and EIS, CR2032 half-cells were prepared according to the electrode fabrication and cell assembly procedure described in Section 2.3. EIS measurements were performed after the formation process over a frequency range of 100 kHz to 0.01 Hz using an AC perturbation amplitude of 5 mV. Spin-polarized DFT calculations were performed with the Vienna Ab initio Simulation Package (VASP Version 6.5.0). The exchange–correlation interaction was treated within the generalized gradient approximation using the Perdew–Burke–Ernzerhof functional. The influence of Cu incorporation on the ZnO electronic structure was evaluated from the calculated band profiles and partial density of states.

### 2.3. Electrode Fabrication

The sodium storage performance of the Zn_1−x_Cu_x_O electrodes was tested in CR2032-type half-cells. To prepare the working electrodes, the active material was mixed with carbon black and polyvinylidene fluoride (PVDF) binder at a mass ratio of 80:10:10. N-methyl-2-pyrrolidone (NMP) was added gradually to obtain a smooth and uniform slurry. The prepared slurry was spread onto copper foil current collectors by the doctor-blade coating method. Copper foil with a thickness of 11 μm and a purity of ≥99.8% (MTI Korea, Seoul, Republic of Korea) was used as the current collector. After coating, the electrode films were dried at 80 °C for 12 h to remove the remaining solvent. The dried films were then pressed under 200 kg cm^−2^ to improve adhesion between the active layer and the copper current collector. Circular working electrode disks with a diameter of 14 mm, corresponding to a geometrical area of 1.54 cm^2^, were punched from the coated copper foil. The active material loading was maintained at approximately 1.30 ± 0.02 mg cm^−2^. All coin cells were assembled inside an argon-filled glovebox. Na^+^ metal foil was used as both the counter electrode and reference electrode. A Whatman GF/D glass microfiber membrane was used as the separator. The electrolyte was composed of 1 M NaClO_4_ dissolved in ethylene carbonate and propylene carbonate with a volume ratio of 1:1. In addition, 5 vol% fluoroethylene carbonate (FEC) was added to improve interfacial stability during cycling.

## 3. Results and Discussions

**Figure 2:**
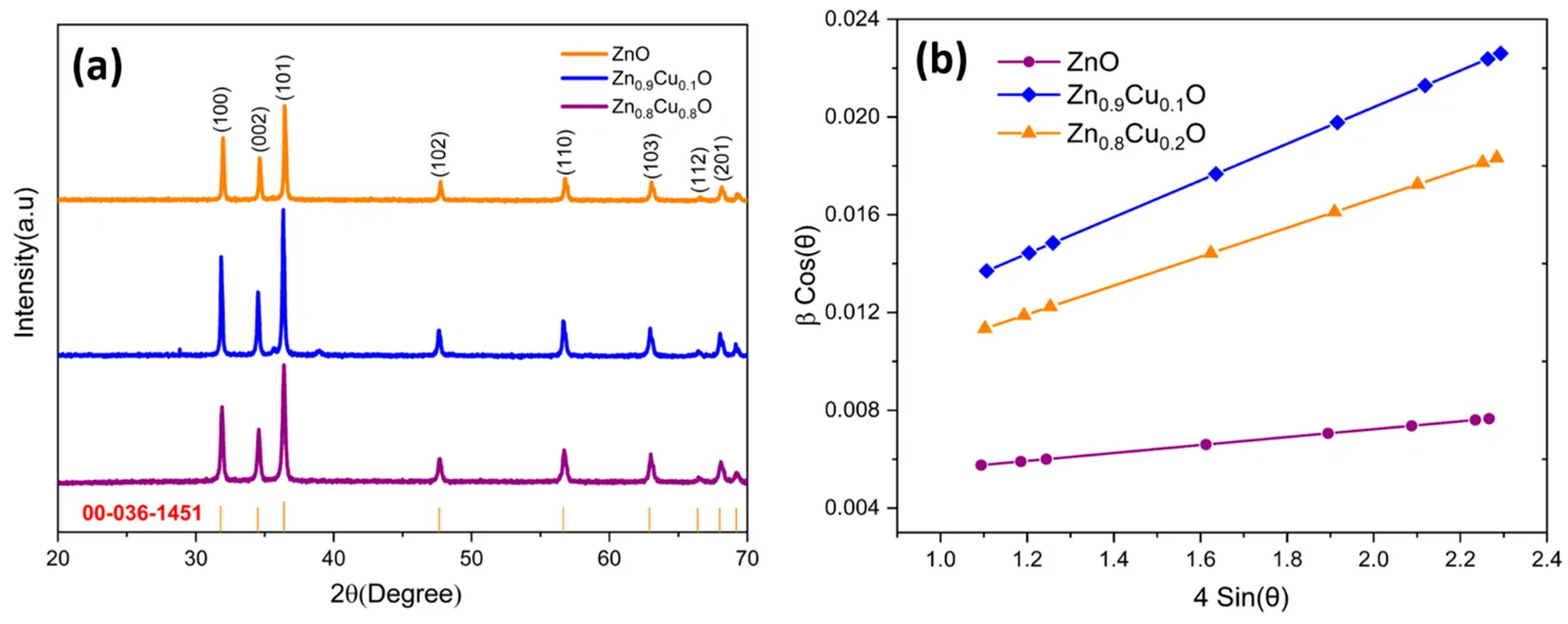
(**a**) XRD patterns of pristine ZnO, Zn_0.9_Cu_0.1_O, and Zn_0.8_Cu_0.2_O nanoparticles; (**b**) corresponding Williamson–Hall plot used for microstrain analysis.

Figure 2a shows the XRD patterns of Zn_1−x_Cu_x_O nanoparticle samples with x = 0.0, 0.1, and 0.2. All samples exhibit a similar diffraction profile, indicating that the main crystal framework is preserved after compositional modification. The major reflections appear at the positions assigned to the hexagonal wurtzite ZnO phase with the P6_3_mc space group, in agreement with JCPDS card No. 36-1451. The relatively sharp diffraction peaks reveal that crystalline ZnO-based nanoparticles were obtained after calcination. Importantly, no extra diffraction signals related to metallic Cu, CuO, or Cu_2_O are observed within the detection limit of XRD, even for the x = 0.2 sample.

The lattice parameters derived from the diffraction data are summarized in Table 1. The obtained lattice constants for Zn_1−x_Cu_x_O, including a = b values in the range of 3.216–3.253 Å and c values in the range of 5.116–5.199 Å, together with the corresponding unit-cell volume of 47.53–45.76 Å^3^, are consistent with previously reported values for doped ZnO systems [22,25]. Compared with pristine ZnO, Cu incorporation produces a clear modification in the lattice dimensions. The Zn_0.9_Cu_0.1_O sample shows the strongest lattice contraction, with a = b decreasing to 3.216 Å, c decreasing to 5.116 Å, and the unit cell volume decreasing to 45.76 Å^3^. This indicates that moderate Cu substitution strongly influences the ZnO crystal framework. When the Cu concentration is increased to x = 0.2, the lattice constants slightly increase to a = b = 3.227 Å and c = 5.168 Å, with a unit cell volume of 46.55 Å^3^. This non-linear change suggests that the structural evolution is influenced by both substitutional incorporation and local strain relaxation at higher Cu concentration.

The structural changes can be related to the comparable ionic radii and different electronic characteristics of Zn^2+^ and Cu^2+^. The ionic radius of Cu^2+^ is close to that of Zn^2+^, which allows Cu^2+^ ions to enter Zn-related lattice positions without destroying the wurtzite structure. However, Cu^2+^ has higher electronegativity than Zn^2+^, which can alter the local metal–oxygen bonding environment. This difference may generate local distortion, modify bond lengths, and induce microstrain in the doped ZnO lattice. Therefore, the changes in lattice constants, unit cell volume, and peak broadening indicate that Cu incorporation affects the internal crystal structure while retaining the overall wurtzite phase. The d_101_ spacing also changes after Cu doping, increasing from 2.4479 Å for pristine ZnO to 2.4574 Å for Zn_0.9_Cu_0.1_O and 2.4616 Å for Zn_0.8_Cu_0.2_O. This variation indicates that Cu incorporation modifies the local interplanar arrangement of the (101) plane. Although the overall unit cell volume of the doped samples is lower than that of pristine ZnO, the increase in d_101_ suggests an anisotropic lattice response, where different crystallographic directions respond differently to Cu substitution, defect formation, and local strain distribution.

The crystallite size was evaluated from the XRD peak broadening. Pristine ZnO shows an average crystallite size of 29.91 nm. After Cu incorporation, the crystallite size decreases significantly to 25.66 nm for Zn_0.9_Cu_0.1_O, indicating that moderate Cu doping effectively restricts crystallite growth. This smaller crystallite size is favorable for sodium-ion storage because it can shorten Na^+^ diffusion pathways and provide more electrochemically accessible active sites. For Zn_0.8_Cu_0.2_O, the crystallite size increases slightly to 28.72 nm, although it remains smaller than that of pristine ZnO. This suggests that excessive Cu incorporation does not further improve crystallite refinement and may lead to partial structural relaxation or the formation of larger coherent crystalline domains.

The Williamson–Hall-derived microstrain further confirms the defect-rich nature of the Cu-doped samples. The microstrain increases from 1.62 × 10^−3^ for pristine ZnO to 7.50 × 10^−3^ for Zn_0.9_Cu_0.1_O, revealing that x = 0.1 Cu incorporation produces the highest lattice distortion. This enhanced strain can arise from local bonding mismatch, lattice distortion, defect formation, and charge redistribution around Cu-related sites. When the Cu content is increased to x = 0.2, the microstrain decreases to 5.91 × 10^−3^, suggesting partial relaxation of the distorted lattice at higher dopant concentrations. However, the strain value remains higher than that of pristine ZnO, confirming that both doped samples possess greater structural disorder than pure ZnO. The dislocation density follows the same trend as the microstrain. It increases from 8.27 × 10^14^ m^−2^ for ZnO to 15.19 × 10^14^ m^−2^ for Zn_0.9_Cu_0.1_O, confirming the formation of a highly defect-enriched structure in the x = 0.1 Cu composition. For Zn_0.8_Cu_0.2_O, the dislocation density decreases to 12.12 × 10^14^ m^−2^, which is still higher than that of pristine ZnO but lower than that of Zn_0.9_Cu_0.1_O. This result indicates that moderate Cu doping generates the most favorable concentration of structural defects, while higher Cu loading partially reduces the defect intensity due to lattice relaxation. These structural features are beneficial for sodium-ion battery performance because reduced crystallite size can shorten Na^+^ diffusion distance, while enhanced strain and defect density can provide additional active sites and promote interfacial reaction kinetics.

**Figure 3:**
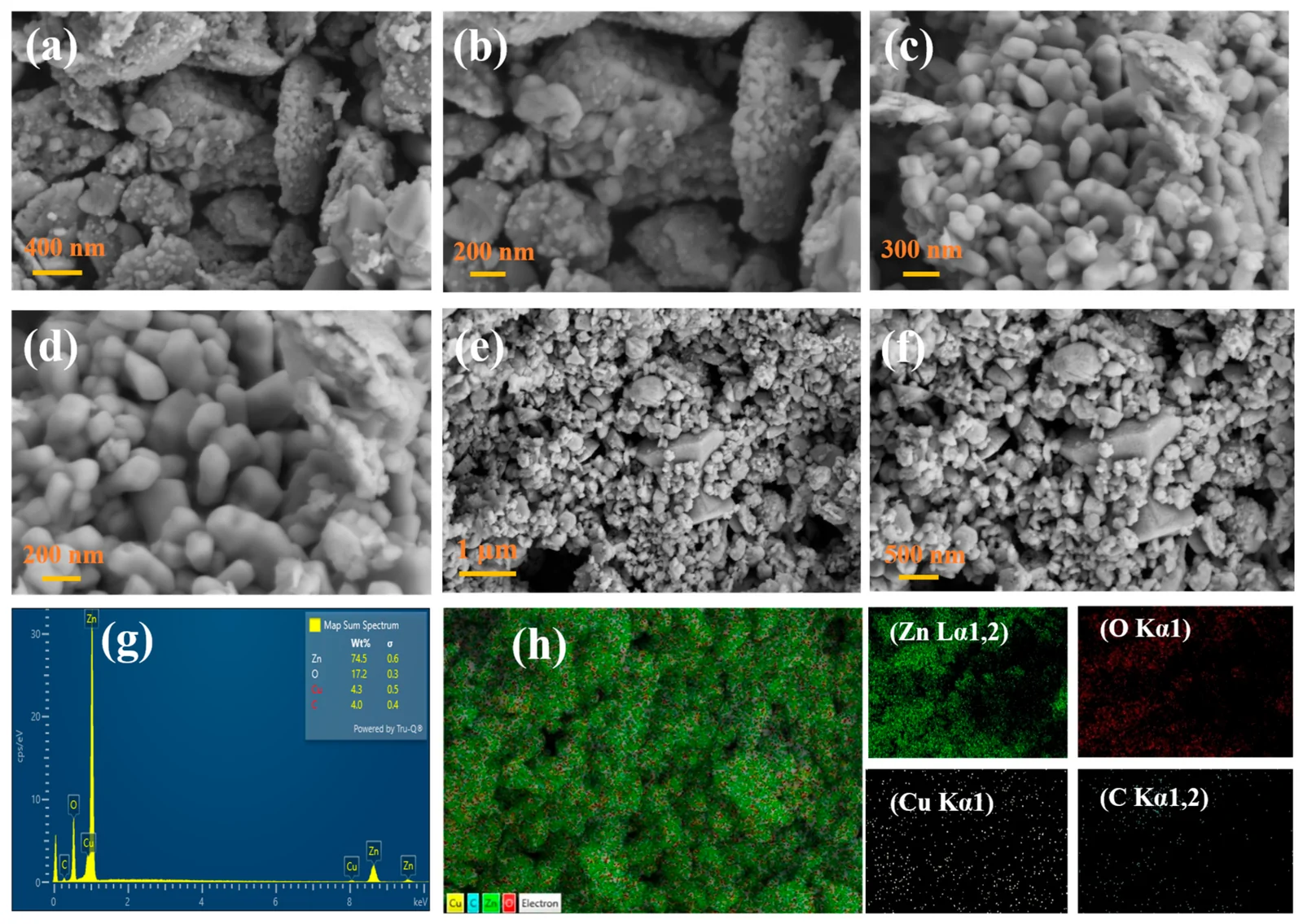
(**a**,**b**) FESEM micrographs of pristine ZnO, (**c**,**d**) Zn_0.9_Cu_0.1_O, and (**e**,**f**) Zn_0.8_Cu_0.2_O nanoparticles; (**g**) EDX spectrum and (**h**) elemental mapping of the Zn_0.9_Cu_0.1_O sample.

The FESEM–EDX results of the Zn_1−x_Cu_x_O samples are summarized in Figure 3a–h. The micrographs are arranged according to composition, where Figure 3a,b represent pristine ZnO, Figure 3c,d correspond to Zn_0.9_Cu_0.1_O, and Figure 3e,f show Zn_0.8_Cu_0.2_O. Pristine ZnO, shown in Figure 3a,b, displays an irregular morphology consisting of non-uniform particles, plate-like fragments, and elongated structures. The particles are strongly agglomerated and show a broad size variation, indicating uncontrolled growth and non-uniform nucleation. Such heterogeneous morphology can be unfavorable for electrochemical applications because large particles and irregular plate-like structures can increase the Na^+^ diffusion length and produce non-uniform electrochemical reaction zones. In addition, the broad particle-size distribution may result in uneven current distribution during charge/discharge, where smaller particles react rapidly, while larger particles respond more slowly. The FESEM images after Cu incorporation show significant modification of the morphology of ZnO, indicating that the dopant plays an important role in controlling particle growth, aggregation behavior, and surface texture during synthesis.

After Cu incorporation at x = 0.1, the morphology changes noticeably, as shown in Figure 3c,d. The Zn_0.9_Cu_0.1_O sample exhibits a more refined and homogeneous particle structure compared with pristine ZnO. The high-magnification image shows rounded nanosized particles with reduced large plate-like features. This indicates that moderate Cu doping suppresses excessive particle growth and promotes a more uniform particle arrangement. Such morphology is advantageous for sodium-ion storage because smaller and more uniformly distributed particles can provide shorter diffusion pathways and more balanced electrochemical activity throughout the electrode. For Zn_0.8_Cu_0.2_O, shown in Figure 3e,f, the particles remain nanosized and more refined than in pristine ZnO; however, the morphology becomes slightly more clustered compared with Zn_0.9_Cu_0.1_O. The particles appear more densely aggregated in some regions, suggesting that excessive Cu incorporation may partially disturb the particle growth behavior. Although Zn_0.8_Cu_0.2_O still shows improved morphology compared with pure ZnO, its particle arrangement is less uniform than that of Zn_0.9_Cu_0.1_O. This observation is consistent with the structural trend obtained from XRD, where Zn_0.8_Cu_0.2_O shows a slightly larger crystallite size and lower microstrain than Zn_0.9_Cu_0.1_O.

**Figure 4:**
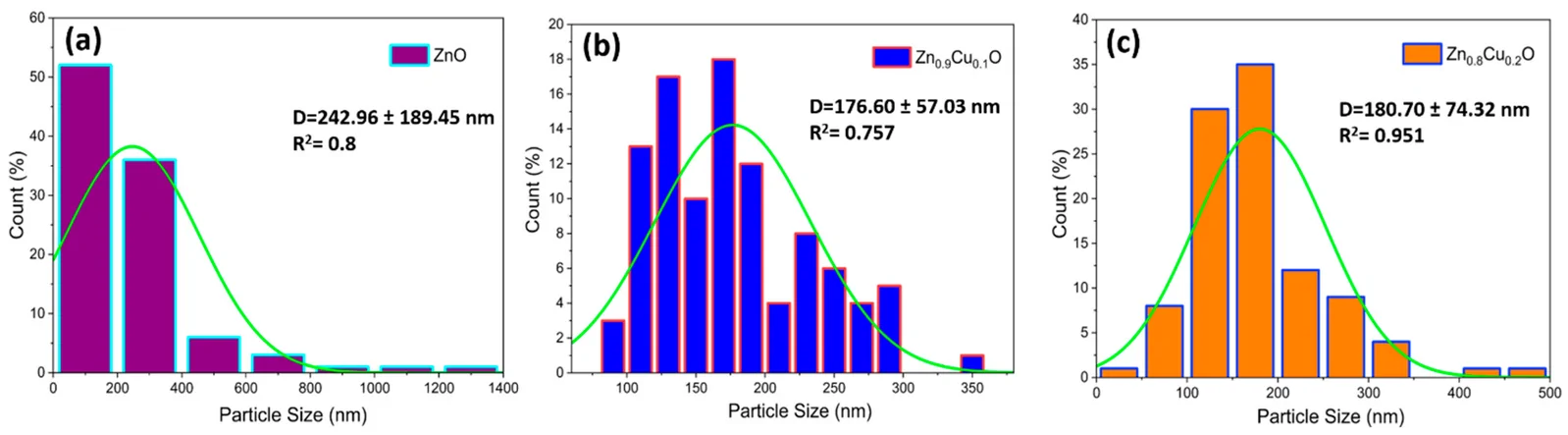
Particle-size distribution histograms of (**a**) ZnO, (**b**) Zn_0.9_Cu_0.1_O, and (**c**) Zn_0.8_Cu_0.2_O obtained from FESEM analysis with log-normal fitting curves.

The particle-size distribution (Figure 4a–c) analysis further supports the FESEM observations. The average particle size decreases from 242.96 nm for pristine ZnO to 176.54 nm for Zn_0.9_Cu_0.1_O, indicating that moderate Cu substitution effectively restricts greater particle growth. For Zn_0.8_Cu_0.2_O, the average particle size slightly increases to 180.23 nm. The large standard deviation of pristine ZnO confirms its broad and non-uniform particle-size distribution, whereas Zn_0.9_Cu_0.1_O shows a narrower size distribution and better morphological uniformity. The reduced particle size and improved size homogeneity of Zn_0.9_Cu_0.1_O can enhance active-material utilization by allowing more uniform electrode/electrolyte interaction and more consistent Na^+^ storage behavior.

The improved morphology of Zn_0.9_Cu_0.1_O can also help reduce polarization during electrochemical cycling. In a highly non-uniform electrode, large particles and small particles do not participate equally in the electrochemical reaction, which can lead to localized stress, sluggish kinetics, and uneven charge storage. In contrast, the more uniform nanosized morphology of Zn_0.9_Cu_0.1_O can promote a more homogeneous reaction distribution. This uniformity is beneficial for conversion/alloying-type ZnO anodes because it can reduce localized mechanical stress and help maintain structural integrity during repeated sodiation/desodiation. Another important feature of Zn_0.9_Cu_0.1_O is the reduction in large bulk-like particles compared with pristine ZnO. Large ZnO particles can contain longer solid-state diffusion paths and may suffer from incomplete utilization during high-current operation. The smaller particle size of Zn_0.9_Cu_0.1_O can shorten the Na^+^ transport distance within the active material and improve rate capability.

The EDX spectrum of Zn_0.9_Cu_0.1_O confirms the presence of Zn, O, and Cu elements. The corresponding elemental composition shows Zn as the dominant element, along with oxygen and a detectable Cu contribution, confirming successful Cu incorporation in the ZnO-based sample. The elemental mapping further demonstrates that Zn, O, and Cu are distributed throughout the selected region without obvious elemental segregation. The homogeneous Cu distribution is important because non-uniform dopant accumulation could form inactive regions or secondary phases, whereas uniform Cu distribution can help maintain consistent electrochemical activity across the electrode.

The particle-size distribution of pristine ZnO and Cu-doped ZnO samples was analyzed from FESEM images. For each sample, 100 particles were measured from representative FESEM micrographs acquired under the same magnification conditions. Particles were selected from different regions of the micrographs to minimize local sampling bias. Only particles with distinguishable boundaries were measured, whereas strongly overlapping or unresolved agglomerates were excluded from individual-particle measurements. The same measurement criterion was applied to all three samples. The measured particle diameters were used to construct the histograms, which were subsequently analyzed using a log-normal distribution. For each sample, the obtained histograms were fitted using a log-normal distribution. Since the particle-size histograms show asymmetric behavior rather than a perfectly normal distribution, the average particle size was calculated from the log-normal fitting parameters rather than from the simple histogram center. For the log-normal fitting, the origin provides the fitted mean of ln(D) (μ) and the fitted mean of the standard deviation of ln(D) (σ). The average particle size and the corresponding standard deviation from the log-normal distribution were calculated using Equations (1) and (2):

(1) $$D_{LN}=e^{\left(\mu +\frac{\sigma^{2}}{2}\right)}$$

(2) $$SD_{LN}=D_{LN}\;\sqrt{e^{\sigma^{2}}-1}$$

In Equations (1) and (2), $D_{LN}$ and $SD_{LN}$ refer to the log-normal average particle size and standard deviation in nm. Using Equations (1) and (2), the average particle size was calculated to be 242.96 ± 189.45 nm for pristine ZnO, 176.60 ± 57.03 nm for Zn_0.9_Cu_0.1_O, and 180.70 ± 74.32 nm for Zn_0.8_Cu_0.2_O. The corresponding coefficients of determination (R^2^) for the log-normal particle-size distributions were 0.8, 0.757, and 0.951 for ZnO, Zn_0.9_Cu_0.1_O, and Zn_0.8_Cu_0.2_O, respectively. Based on the fitted distributions, Cu incorporation significantly reduces the particle size of ZnO, with the x = 0.1 composition showing the smallest average particle size and the narrowest size distribution. The pristine ZnO sample in Figure 4a shows the broadest particle-size distribution among the three samples. The distribution contains a mixture of smaller particles and much larger particles/agglomerates, producing a long tail toward higher particle sizes. This broad distribution is consistent with the FESEM images, where pristine ZnO exhibits irregular particles, plate-like fragments, and elongated structures. The very high standard deviation of 189.45 nm confirms that pristine ZnO has poor particle-size uniformity. Such a wide distribution can be unfavorable for electrochemical storage because particles of very different sizes may not participate uniformly during sodiation/desodiation. Smaller particles can react quickly, while larger particles may show slower Na+ diffusion and delayed electrochemical response.

After Cu incorporation at x = 0.1, the particle-size distribution becomes much narrower, as shown in Figure 4b. The average particle size decreases from 242.96 nm for ZnO to 176.60 nm for Zn_0.9_Cu_0.1_O, while the standard deviation decreases sharply from 189.45 to 57.03 nm. This large reduction in the standard deviation shows that the x = 0.1 sample has a more uniform particle population. The absence of a strong high-size tail suggests that moderate Cu incorporation suppresses excessive particle growth and reduces the formation of large irregular ZnO particles. This agrees with the FESEM images, where Zn_0.9_Cu_0.1_O shows a more refined and rounded nanosized morphology. For Zn_0.8_Cu_0.2_O, shown in Figure 4c, the particle-size distribution remains much narrower than that of pristine ZnO, but it becomes slightly broader than that of Zn_0.9_Cu_0.1_O. The calculated average particle size is 180.70 ± 74.32 nm. Although this value is still much lower than that of pristine ZnO, it is slightly higher than that of Zn_0.9_Cu_0.1_O. The higher standard deviation compared with Zn_0.9_Cu_0.1_O indicates that increasing the Cu content to x = 0.2 does not further improve particle-size control. Instead, the broader distribution suggests the formation of more clustered or larger secondary particles at higher Cu concentrations.

The narrower distribution of Zn_0.9_Cu_0.1_O is important because a more uniform particle population can support more homogeneous electrochemical reactions across the electrode. In contrast, the broad distribution of pristine ZnO can lead to uneven reaction kinetics, where larger particles contribute less effectively during fast charge/discharge. The reduction in particle size is beneficial for sodium-ion storage because smaller particles provide shorter solid-state Na^+^ diffusion pathways. In large ZnO particles or agglomerates, inner regions may be less accessible during cycling, especially at higher current densities. By contrast, the refined particle size of Zn_0.9_Cu_0.1_O can improve active-material utilization and reduce kinetic limitations.

**Figure 5:**
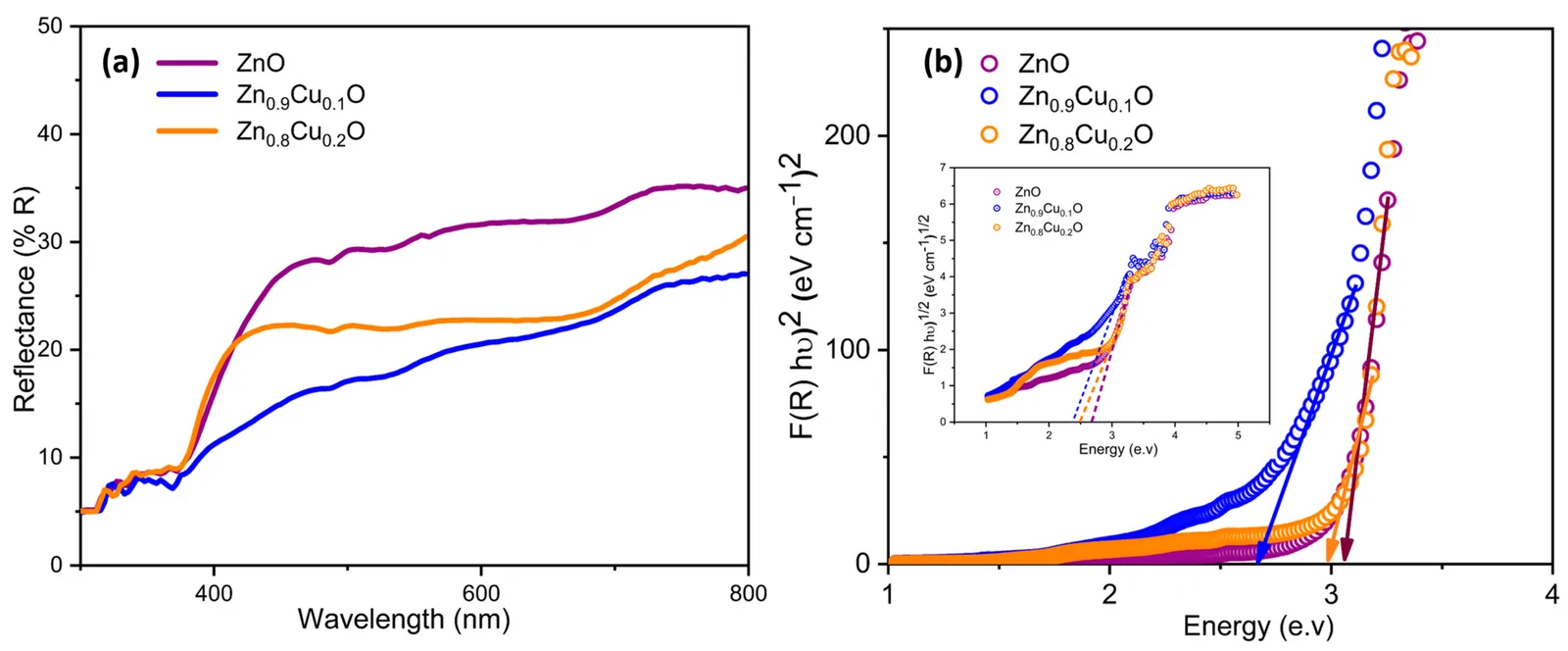
Optical response of Zn_1−x_Cu_x_O nanoparticles with x = 0.0, 0.1, and 0.2: (**a**) diffuse reflectance spectra and (**b**) corresponding direct Tauc plots obtained from the Kubelka–Munk-transformed data, with the corresponding indirect Tauc plots shown in the inset.

Diffuse reflectance spectroscopy (DRS) was used to examine the optical absorption behavior and band structure modification of the Zn_1−x_Cu_x_O (x = 0, 0.1, 0.2) nanoparticles (Figure 5a,b). The reflectance spectra were converted into the Kubelka–Munk function using Equation (3):

(3) $$F(R)=\frac{(1-R{)}^{2}}{2R}$$

where R is the diffuse reflectance expressed as a fraction [26]. The optical band gap was then obtained from the Tauc relation ${\left[F(R)\;h\nu \right]}^{m}=A(h\nu -E_{g})$, where hν is the photon energy, A is the proportionality constant, and E_g_ is the optical band gap. For a direct allowed transition, m = 2, and therefore, ${\left[F(R)\;h\nu \right]}^{2}$ was plotted against hν; for an indirect allowed transition, m = 1/2, and ${\left[F(R)\;h\nu \right]}^{1/2}$ was plotted against hν. The band gap values were determined by linear regression of the fundamental absorption-edge region and extrapolation of the fitted line to the photon-energy axis. To distinguish the fundamental absorption edge from defect-related or sub-band gap absorption, only the steep linear portion of the main absorption edge was used for the Tauc extrapolation, while the lower-energy absorption-tail region was excluded from the fitting [27].

The diffuse reflectance spectra show that pristine ZnO exhibits relatively high reflectance in the visible region, with an average reflectance of 29.28% between 400 and 700 nm. After Cu incorporation, the reflectance decreases markedly, indicating stronger optical absorption. The Zn_0.9_Cu_0.1_O sample shows the lowest visible-region reflectance of 18.36%, while Zn_0.8_Cu_0.2_O shows a partially recovered reflectance of 22.31%. The corresponding average Kubelka–Munk response follows the opposite trend, increasing from 0.886 for pristine ZnO to 1.909 for Zn_0.9_Cu_0.1_O and then decreasing to 1.357 for Zn_0.8_Cu_0.2_O. This indicates that the optical absorption is maximized at the intermediate Cu concentration, rather than increasing continuously with Cu content.

Pristine ZnO shows a direct band gap of 3.08 eV, which is consistent with the typical wide-band-gap nature of ZnO. After 10% Cu incorporation, the band gap decreases strongly to 2.66 eV, corresponding to an absorption-edge shift from 402.60 to 466.17 nm. This large red shift of 63.57 nm confirms that Cu incorporation at x = 0.1 significantly lowers the optical transition energy. The indirect band gap follows the same tendency, decreasing from 2.67 eV for ZnO to 2.37 eV for Zn_0.9_Cu_0.1_O. When the Cu concentration is increased to x = 0.2, the direct band gap increases again to 2.99 eV, while the indirect band gap increases to 2.49 eV. This partial recovery of the band gap indicates that the optical response of Zn_1−x_Cu_x_O is not governed simply by the amount of Cu added. Instead, the results suggest that 10% Cu substitution produces the strongest band-edge modification, whereas a higher Cu content partially weakens the band-gap-narrowing effect. Therefore, Zn_0.9_Cu_0.1_O shows the most pronounced red shift and the strongest visible-light absorption among the three samples.

The strong band gap narrowing observed for Zn_0.9_Cu_0.1_O can be attributed to Cu-induced modification of the ZnO electronic structure. Cu ions can introduce localized electronic states within or near the band edges of ZnO. In addition, interaction between Cu 3d states and O 2p states can modify the valence-band region and reduce the energy required for optical excitation. These Cu-related localized states can act as intermediate electronic levels, allowing lower-energy photons to contribute to electronic transitions. As a result, the absorption edge shifts toward the visible region and the optical band gap decreases. The increase in band gap at x = 0.2 suggests a change in the dominant optical mechanism at higher Cu concentrations. At moderate doping, Cu ions are more effectively incorporated into the ZnO framework and strongly perturb the band-edge structure. However, at higher Cu contents, local defect compensation, Cu-related defect complexes, partial dopant clustering below the XRD detection limit, or structural relaxation may reduce the effectiveness of isolated Cu-ZnO electronic interactions. These effects can partially restore the optical transition energy, producing the observed blue-shift behavior from 2.66 to 2.99 eV. The Urbach energy and steepness were also evaluated to understand the degree of band-tail formation. Urbach energy and steepness were obtained from Equations (4) and (5).

(4) $$\ln\left[F\left(R\right)\right]=\ln\left(F_{o}\right)+\frac{h\nu }{E_{U}}$$

(5) $$S=\frac{K_{BT}}{E_{U}}$$

The Urbach energy (E_U_) is calculated from the reciprocal slope of the linear region of the $\ln\left[F\left(R\right)\right]\;$ versus hν plot. The corresponding coefficients of determination (R^2^) were 0.9903, 0.9905, and 0.9984 for ZnO, Zn_0.9_Cu_0.1_O, and Zn_0.8_Cu_0.2_O, respectively. The pristine ZnO sample shows an Urbach energy of 0.505 ± 0.009 eV and a steepness parameter of 0.0512. After Cu doping, the Urbach energy increases to 0.862 ± 0.005 eV for Zn_0.9_Cu_0.1_O and 0.529 ± 0.032 for Zn_0.8_Cu_0.2_O, while the steepness parameter decreases to 0.0119 and 0.0127, respectively. The increase in E_U_ for Zn_0.9_Cu_0.1_O indicates enhanced band-edge disorder after moderate Cu incorporation, whereas the lower value for Zn_0.8_Cu_0.2_O indicates a non-monotonic evolution of the band-tail states with increasing Cu content. The highest Urbach energy for Zn_0.9_Cu_0.1_O agrees with its strongest optical absorption, lowest reflectance, and largest band gap narrowing. In contrast, Zn_0.8_Cu_0.2_O still shows enhanced band-tail disorder compared with pristine ZnO, but its partial band gap recovery suggests that excessive Cu incorporation changes the defect/electronic balance and reduces the effectiveness of band gap narrowing. From the viewpoint of SIB performance, Zn_0.9_Cu_0.1_O exhibits the strongest band gap narrowing among the investigated compositions. DFT further indicates Cu-induced electronic-state modification near the Fermi level, while EIS shows the most favorable charge-transfer behavior for the same composition.

**Figure 6:**
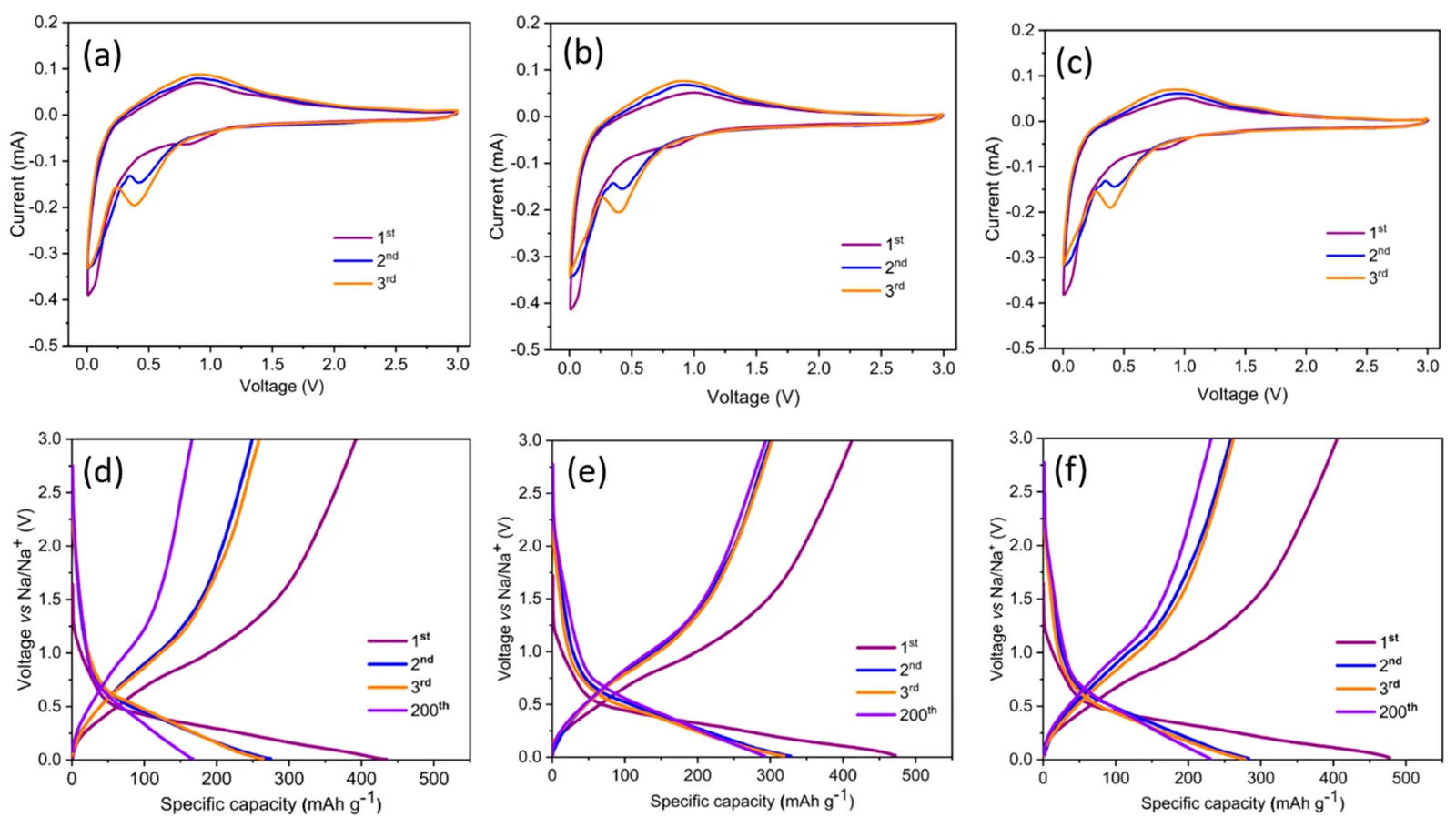
CV curves recorded at 0.1 mV s^−1^ for (**a**) Pure ZnO, (**b**) 10% ZnO, and (**c**) 20% ZnO; galvanostatic charge–discharge profiles of (**d**) Pure ZnO, (**e**) 10% ZnO, and (**f**) 20% ZnO.

Cyclic voltammetry was carried out to clarify the redox processes involved during Na^+^ storage in the Zn_1−x_Cu_x_O electrodes. The measurements were performed between 0.01 and 3.0 V at a scan rate of 0.1 mV s^−1^, and the obtained curves are shown in Figure 6a. In the first cathodic sweep, a broad reduction response appears, which is mainly associated with initial electrode activation, electrolyte decomposition, and formation of the solid–electrolyte interphase layer [28]. The weakening of this irreversible contribution in the following cycles indicates the stabilization of the electrolyte/electrode interface. After the first activation cycle, the second and third cathodic scans show reduction peaks at 0.42 and 0.38–0.39 V, respectively. These peaks are attributed to the conversion reaction of ZnO into metallic Zn accompanied by the formation of Na_2_O, followed by partial Na-Zn alloying at lower potentials [29]. During the reverse anodic scan, oxidation peaks appear in the range of 0.88–0.93 V, corresponding to dealloying of Na-Zn phases and partial reoxidation of metallic Zn during sodium extraction [15]. The presence of these cathodic/anodic peak pairs confirms that sodium storage in the ZnO-based electrodes proceeds through a combined conversion/alloying mechanism.

For pristine ZnO, the third-cycle anodic peak appears at 0.88 V with a peak current of 0.08745 mA, while the cathodic conversion peak appears at 0.383 V with a peak current of −0.19531 mA. The peak separation ∆E_p_ is calculated to be 0.499 V, indicating relatively high polarization. Although pristine ZnO exhibits a large CV loop area of 0.25764 mA V in the third cycle, the broad peak shape and relatively larger polarization suggest sluggish charge-transfer kinetics and significant hysteresis during sodium insertion/extraction. After Cu substitution, the Zn_0.9_Cu_0.1_O electrode exhibits a more favorable CV response. In the third cycle, the anodic peak is located at 0.891 V with a peak current of 0.07602 mA, while the cathodic conversion peak appears at 0.394 V with a stronger cathodic current of −0.20483 mA. The corresponding peak separation is 0.497 V, which is lower than that of pristine ZnO and Zn_0.8_Cu_0.2_O. This smaller ∆E_p_ indicates reduced polarization and faster redox kinetics. In addition, Zn_0.9_Cu_0.1_O exhibits a third-cycle CV loop area of 0.24440 mA V and a redox-region area of 0.13259 mA V, which are higher than those of Zn_0.8_Cu_0.2_O. These results show that 10% Cu incorporation improves the useful redox response while maintaining sufficient electrochemically active Zn sites. For Zn_0.8_Cu_0.2_O, the third-cycle anodic and cathodic peaks are located at 0.931 and 0.389 V, respectively, giving a larger ∆E_p_ of 0.543 V. Although this electrode shows the highest second-to-third cycle overlap index of 0.943, its anodic peak current decreases to 0.06975 mA, and its third-cycle loop area decreases to 0.22332 mA V. This indicates that excessive Cu substitution improves cycle-to-cycle stability but reduces the overall redox activity. The decrease in peak current and CV area can be attributed to the reduced concentration of electrochemically active Zn species participating in conversion/alloying reactions.

The superior electrochemical behavior of the 10% Cu-doped ZnO electrode can be credited to the beneficial role of Cu incorporation in improving the electronic and interfacial properties of ZnO. Cu substitution can enhance electronic conductivity, modify the ZnO electronic structure through Cu 3d-O 2p interaction, improve charge mobility, and generate more favorable charge-transfer pathways. In addition, Cu-related reduced species formed during electrochemical activation may act as conductive domains, facilitating electron transport within the electrode matrix. Cu doping at 10% may also help buffer structural stress during repeated sodiation/desodiation, thereby improving electrochemical reversibility. However, excessive Cu substitution reduces the concentration of Zn-related active sites and weakens the conversion/alloying contribution, which explains the lower CV area and smaller peak current observed for Zn_0.8_Cu_0.2_O. The kinetic improvement suggested by CV analysis is further supported by the EIS results. The Zn_0.9_Cu_0.1_O electrode exhibits the lowest fitted specific charge-transfer resistance (R_ct_), confirming faster interfacial charge transfer and improved electrode reaction kinetics.

The charge–discharge response of the Zn_1−x_Cu_x_O (x = 0.0, 0.1, and 0.2) electrodes was further examined under galvanostatic conditions. The cells were cycled between 0.01 and 3.0 V at a current density of 0.1 A g^−1^ for 200 cycles, and the corresponding profiles are presented in Figure 6d–f. The voltage plateaus observed in the galvanostatic curves are consistent with the redox features observed in the CV profiles, confirming that both measurements originate from the same multistep sodium storage process involving SEI formation, conversion reaction, alloying, and dealloying. Specifically, the sloping discharge region around 0.4–0.7 V agrees well with the cathodic conversion peaks observed near 0.41–0.38 V in the CV curves, while the charge plateau around 0.8–1.0 V corresponds to the anodic peaks near 0.89–0.93 V. This agreement confirms that the capacity contribution originates from reversible ZnO-based conversion/alloying reactions rather than only capacitive or irreversible side reactions.

During the first discharge, the pristine ZnO, Zn_0.9_Cu_0.1_O, and Zn_0.8_Cu_0.2_O electrodes deliver initial discharge capacities of 435.24, 471.96, and 477.54 mAh g^−1^, respectively. After the first cycle, a clear capacity decrease occurs in the second cycle due to irreversible sodium consumption and SEI stabilization. The second-cycle discharge capacities of ZnO, Zn_0.9_Cu_0.1_O, and Zn_0.8_Cu_0.2_O are 274.52, 327.74, and 283.44 mAh g^−1^, respectively. The capacity loss from the first to the second cycle is 36.93% for pristine ZnO, 30.56% for Zn_0.9_Cu_0.1_O, and 40.65% for Zn_0.8_Cu_0.2_O, indicating that the Zn_0.9_Cu_0.1_O electrode undergoes the lowest activation-related capacity decay.

The third-cycle discharge capacities further confirm the superior reversibility of the Zn_0.9_Cu_0.1_O electrode. ZnO, Zn_0.9_Cu_0.1_O, and Zn_0.8_Cu_0.2_O deliver third-cycle discharge capacities of 264.28, 319.16, and 278.23 mAh g^−1^, respectively. Although Zn_0.8_Cu_0.2_O exhibits the highest initial discharge capacity, its larger irreversible capacity loss suggests that a considerable part of the first-cycle capacity originates from irreversible side reactions rather than reversible sodium storage. After 200 cycles, Zn_0.9_Cu_0.1_O maintains the highest reversible capacity of 292.58 mAh g^−1^, which is significantly higher than that of pristine ZnO (167.13 mAh g^−1^) and Zn_0.8_Cu_0.2_O (230.14 mAh g^−1^). The capacity retention of Zn_0.9_Cu_0.1_O relative to the third cycle reaches 91.67%, compared with 63.24% for ZnO and 82.72% for Zn_0.8_Cu_0.2_O. This result demonstrates that moderate Cu incorporation greatly improves the cycling stability and reversibility of ZnO during repeated sodiation/desodiation.

The improved GCD performance of Zn_0.9_Cu_0.1_O can be associated with the favorable effect of moderate Cu incorporation within the ZnO framework. At a moderate Cu concentration, Cu–O–Zn interaction can promote local electronic transport and facilitate faster charge transfer during sodiation/desodiation. During electrochemical activation, Cu-containing species may also contribute to the formation of conductive pathways inside the electrode, helping to maintain electrical contact between active particles during repeated volume changes. This effect is particularly important for conversion-type ZnO anodes, where pulverization and loss of electrical connectivity commonly cause capacity fading. Therefore, Zn_0.9_Cu_0.1_O exhibits lower activation-related capacity loss, stronger reversible capacity after stabilization, and better long-term capacity retention. However, excessive Cu substitution at x = 0.2 reduces the concentration of Zn-centered active sites available for conversion/alloying reactions, resulting in lower reversible capacity despite improved stability compared with pristine ZnO.

**Figure 7:**
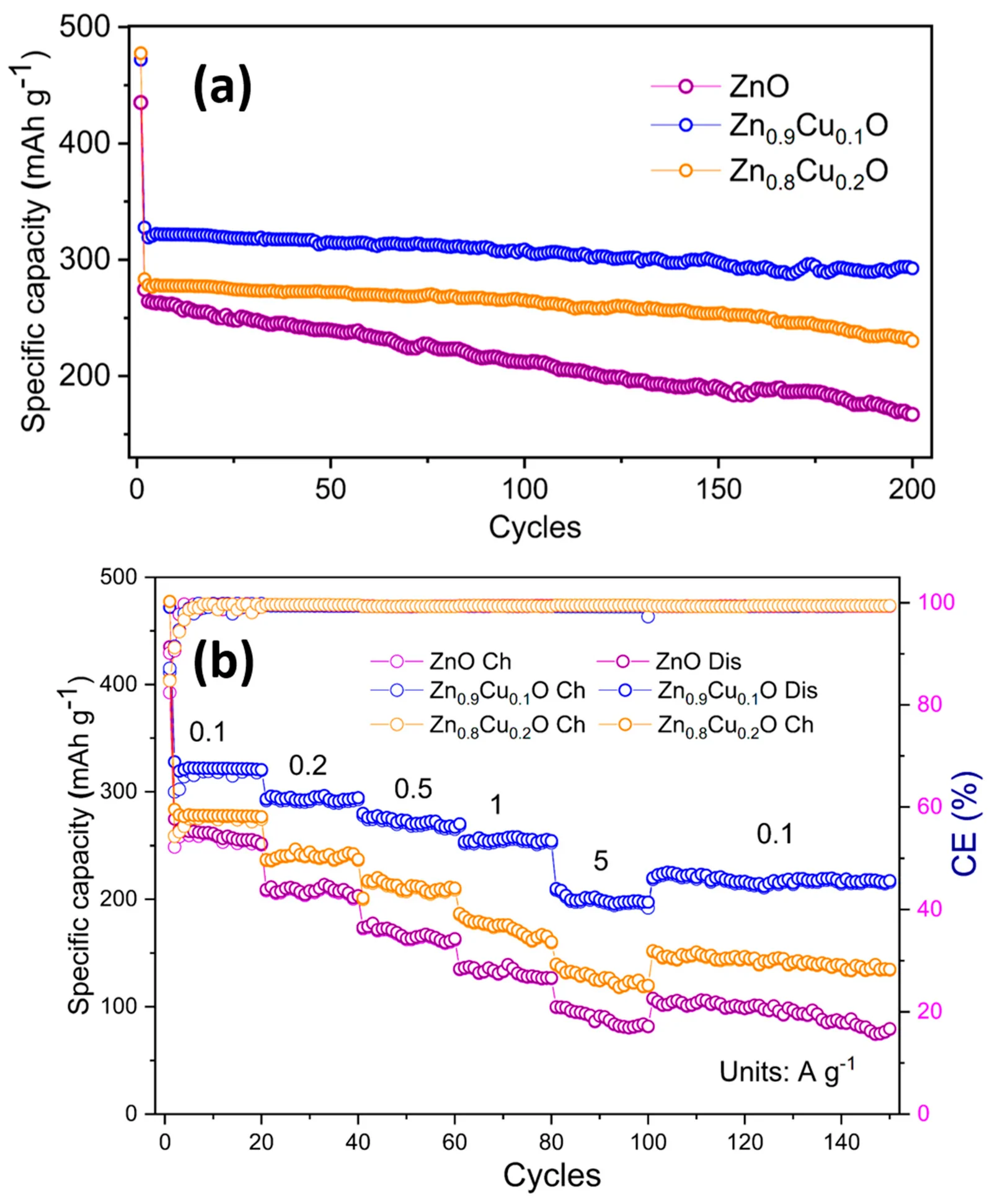
(**a**) Zn_1−x_Cu_x_O (x = 0, 0.1, 0.2) cycling performance comparison at 0.1 A g^−1^ current density; (**b**) Zn_1−x_Cu_x_O (x = 0, 0.1, 0.2) rate performance at various current densities.

The cycling stability of all electrodes was examined for 200 cycles at 0.1 A g^−1^, as presented in Figure 7a. For pristine ZnO, a large capacity drop occurs during the initial cycles. The discharge capacity changes from 435.24 mAh g^−1^ in the first cycle to 274.52 mAh g^−1^ in the second cycle and 264.28 mAh g^−1^ in the third cycle. This large early capacity loss is mainly associated with irreversible Na^+^ consumption, SEI formation, and structural rearrangement during the first sodiation/desodiation process. During prolonged cycling, the capacity of pristine ZnO continues to decrease gradually, reaching only 167.13 mAh g^−1^ after 200 cycles. The retention calculated from the third cycle is 63.24%, indicating poor long-term reversibility and progressive degradation caused by repeated conversion/alloying reactions. The Zn_0.9_Cu_0.1_O electrode shows a distinctly improved cycling response. Although its first discharge capacity is 471.96 mAh g^−1^, the capacity stabilizes rapidly after the initial activation cycles and remains much higher than that of pristine ZnO throughout the entire test. The second and third discharge capacities are 327.74 and 319.16 mAh g^−1^, respectively, indicating improved reversibility after SEI stabilization. Importantly, Zn_0.9_Cu_0.1_O maintains 308.86 mAh g^−1^ at the 100th cycle and still delivers 292.58 mAh g^−1^ after 200 cycles. This corresponds to a high capacity retention of 91.67% relative to the third cycle, with an average fading rate of only 0.135 mAh g^−1^ per cycle from the third to the 200th cycle. These results demonstrate that moderate Cu substitution strongly improves the cycling stability and reversible sodium storage capability of ZnO. The Zn_0.8_Cu_0.2_O electrode delivers the highest initial discharge capacity of 477.54 mAh g^−1^, but this high first-cycle value is followed by a pronounced decrease to 283.44 mAh g^−1^ in the second cycle and 278.23 mAh g^−1^ in the third cycle. After 200 cycles, Zn_0.8_Cu_0.2_O retains 230.14 mAh g^−1^, corresponding to 82.72% retention relative to the third cycle. Although this performance is better than that of pristine ZnO, it is lower than that of Zn_0.9_Cu_0.1_O. The larger first-cycle loss and lower long-term capacity suggest that excessive Cu substitution increases irreversible activation and reduces the contribution of Zn-centered active sites involved in reversible conversion/alloying reactions.

The rate response of all prepared electrodes was tested by increasing the current density stepwise from 0.1 to 5.0 A g^−1^ and then returning it to 0.1 A g^−1^, as shown in Figure 7b. For the pristine ZnO electrode, the capacity decreases continuously as the applied current becomes higher. The discharge capacities are 435.24, 209.10, 173.76, 135.27, and 99.93 mAh g^−1^ at 0.1, 0.2, 0.5, 1.0, and 5.0 A g^−1^, respectively. After the current density is switched back to 0.1 A g^−1^, the recovered capacity is only 107.79 mAh g^−1^. This weak recovery indicates that pristine ZnO cannot tolerate high-current cycling effectively, mainly because of slow charge-transfer kinetics and limited reversibility after fast sodiation/desodiation. In comparison, the Zn_0.9_Cu_0.1_O electrode exhibits the most favorable rate capability among the investigated electrodes. It delivers discharge capacities of 471.96, 293.13, 279.78, 269.96, and 209.96 mAh g^−1^ at 0.1, 0.2, 0.5, 1.0, and 5.0 A g^−1^, respectively. Even at the high current density of 5.0 A g^−1^, Zn_0.9_Cu_0.1_O maintains 209.96 mAh g^−1^, which is 2.10 times higher than that of pristine ZnO. After the current density is returned to 0.1 A g^−1^, the electrode recovers to 219.96 mAh g^−1^, confirming better tolerance to fast charge–discharge operation. The Zn_0.8_Cu_0.2_O electrode delivers 477.54, 236.98, 201.24, 186.71, and 139.59 mAh g^−1^ at 0.1, 0.2, 0.5, 1.0, and 5.0 A g^−1^, respectively. Although Zn_0.8_Cu_0.2_O shows the highest initial discharge capacity at 0.1 A g^−1^, its capacity decreases more strongly at higher current densities compared with Zn_0.9_Cu_0.1_O. At 5.0 A g^−1^, the capacity of Zn_0.8_Cu_0.2_O is 139.59 mAh g^−1^, which is much lower than that of Zn_0.9_Cu_0.1_O. This shows that excessive Cu substitution is less effective under fast Na^+^ insertion/extraction conditions.

A clearer comparison can be obtained by considering the capacity retention from 0.2 to 5.0 A g^−1^. Pristine ZnO retains only 47.79% of its 0.2 A g^−1^ capacity at 5.0 A g^−1^, while Zn_0.8_Cu_0.2_O retains 58.91%. In contrast, Zn_0.9_Cu_0.1_O retains 71.63%, demonstrating the best high-rate endurance. The smaller capacity loss of Zn_0.9_Cu_0.1_O under increasing current density indicates a faster reaction response and lower kinetic limitation during rapid sodiation/desodiation. The rate performance results provide different information from the long-term cycling test. While cycling performance reflects capacity preservation during repeated operation, the rate test evaluates how efficiently the electrode can respond when the reaction time becomes shorter at high current density. The superior high-rate response of Zn_0.9_Cu_0.1_O suggests that the x = 0.1 composition provides a more efficient transport pathway for fast Na^+^/electron movement. This behavior is consistent with the lower polarization observed in CV analysis and can be further supported by the lower specific charge-transfer resistance from EIS. Therefore, the x = 0.1 composition exhibits improved capacity retention during cycling as well as a favorable dynamic response under high-current conditions.

**Figure 8:**
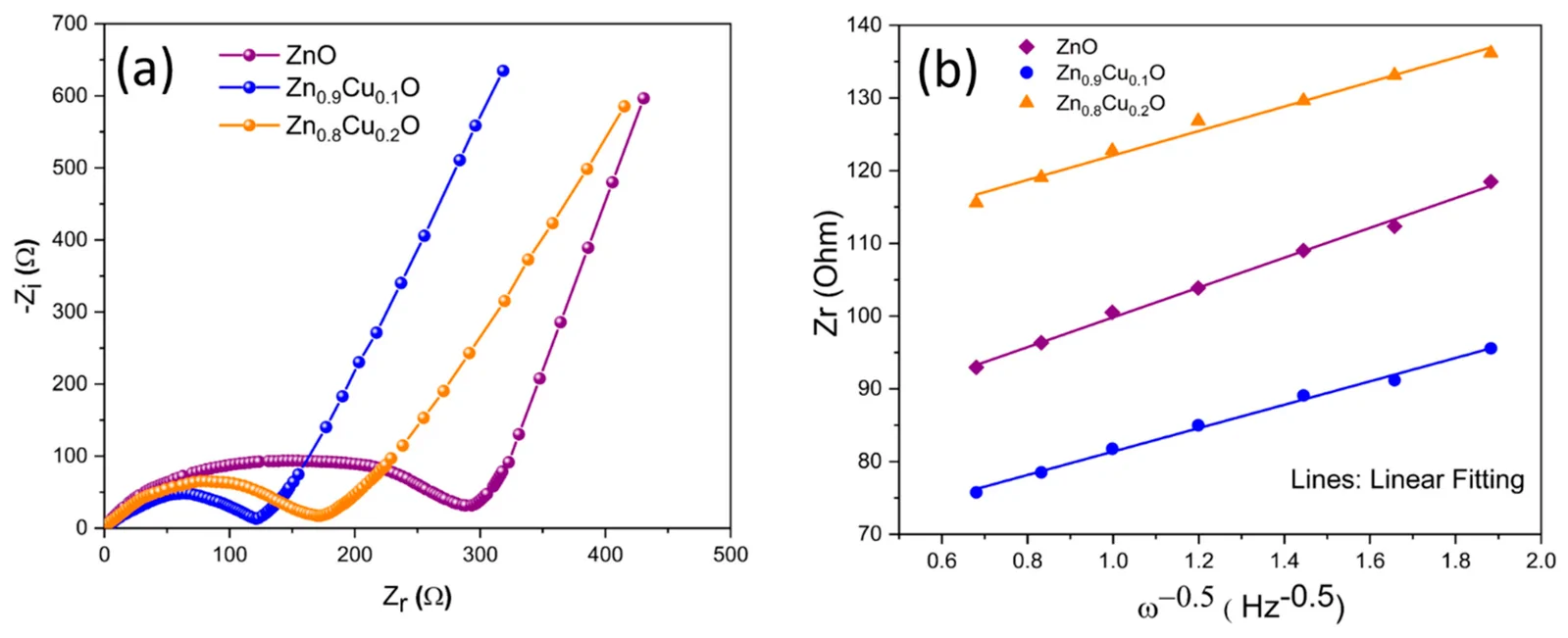
(**a**) Electrochemical impedance analysis of ZnO, Zn_0.9_Cu_0.1_O, and Zn_0.8_Cu_0.2_O electrodes: (**a**) Nyquist plots and (**b**) fitting of (Z’) versus in the low-frequency region for Warburg coefficient estimation.

Electrochemical impedance analysis was employed to gain deeper insight into the kinetic role of Cu substitution in ZnO electrodes during Na^+^ storage. The Nyquist plots of ZnO [30], Zn_0.9_Cu_0.1_O, and Zn_0.8_Cu_0.2_O are displayed in Figure 8a, and the corresponding linear fitting of Z′ as a function of ω^−1/2^ in the diffusion-controlled region is presented in Figure 8b. From the plot, the values of solution resistance R_s_ for ZnO, Zn_0.9_Cu_0.1_O, and Zn_0.8_Cu_0.2_O are found to be 0.74, 1.87, and 1.26 Ω, respectively. Since these values are relatively small, their variation is mainly attributed to cell assembly factors such as electrolyte wetting, separator contact, and electrode/current collector contact rather than the intrinsic reaction kinetics of the active material. Therefore, the specific charge-transfer resistance provides a more meaningful comparison of the interfacial reaction behavior. The pristine ZnO electrode exhibits the largest R_ct_ value of 467.76 Ω cm^2^, indicating sluggish interfacial charge transfer. After introducing Cu, the R_ct_ is markedly reduced to 185.15 Ω cm^2^ for Zn_0.9_Cu_0.1_O, demonstrating that moderate Cu incorporation greatly facilitates the electrode/electrolyte reaction. When the Cu content is further increased to x = 0.2, the Rct increases to 256.44 Ω cm^2^, but it remains much lower than that of pristine ZnO. This result suggests that 10% Cu substitution improves the interfacial kinetics of ZnO, while an excessive Cu content partially weakens the kinetic advantage.

To further compare the ease of charge transport, the charge-transfer conductance was calculated using G_ct_ = 1/R_ct_. The G_ct_ value increases from 3.29 mS for ZnO to 8.32 mS for Zn_0.9_Cu_0.1_O, followed by a slight decrease to 6.01 mS for Zn_0.8_Cu_0.2_O. The higher Gct of the Cu-doped electrodes confirms that Cu incorporation promotes faster charge transfer. Among the three electrodes, Zn_0.9_Cu_0.1_O shows the highest G_ct_, indicating the most favorable interfacial reaction pathway. The Na^+^ diffusion behavior was evaluated from the Warburg coefficient obtained from the low-frequency linear relationship between Z′ and ω^−1/2^:

Z^′^ = R_s_ + R_ct_ + σω^−1/2^ (6)

where σ is the Warburg coefficient [31]. The fitted σ values are 20.50 ± 0.57, 16.09 ± 0.51, and 16.83 ± 0.87 Ω s^−1/2^ for ZnO, Zn_0.9_Cu_0.1_O, and Zn_0.8_Cu_0.2_O, respectively. Since a smaller σ indicates a lower diffusion resistance, the Cu-doped electrodes possess more favorable Na^+^ transport behavior than pristine ZnO. The Na^+^ diffusion coefficient was calculated using Equation (7) [31]:

(7) $$D_{{Na}^{+}}=\frac{R^{2}T^{2}}{{2n}^{4}F^{4}A^{2}\sigma^{2}C_{{Na}^{+}}^{2}}$$

where C_Na+_ is the Na^+^ concentration, R is the gas constant, and A is the electrode area. The calculated D_Na+_ values are 3.56 × 10^−11^, 5.77 × 10^−11^, and 5.28 × 10^−11^ cm^2^ s^−1^ for Zn_0.9_Cu_0.1_O and Zn_0.8_Cu_0.2_O, respectively. The increase in D_Na+_ after Cu incorporation indicates that Cu substitution improves Na^+^ diffusion within the ZnO electrode. The highest D_Na+_ value obtained for Zn_0.9_Cu_0.1_O reveals that moderate Cu doping provides the most efficient ion-transport pathway. Although Zn_0.8_Cu_0.2_O also exhibits a higher D_Na+_ than pristine ZnO, its slightly lower value compared with Zn_0.9_Cu_0.1_O suggests that excessive Cu incorporation may introduce additional structural disorder, or less favorable diffusion channels.

The exchange current density and apparent standard rate constant were also calculated to quantitatively assess interfacial reaction kinetics:

(8) $$J_{0}=\frac{RT}{nFR_{ct}A}$$

(9) $$K^{0}=\frac{RT}{n^{2}F^{2}AR_{ct}C_{{Na}^{+}}}$$

The J_0_ values are 54.93, 138.76, and 100.19 μA cm^−2^ for ZnO, Zn_0.9_Cu_0.1_O, and Zn_0.8_Cu_0.2_O, respectively. In the same order, the K_0_ values are 5.69 × 10^−7^, 1.44 × 10^−6^, and 1.04 × 10^−6^ cm s^−1^. These values clearly show that Cu substitution accelerates the interfacial electrochemical reaction. The Zn_0.9_Cu_0.1_O electrode delivers the highest J_0_ and K^0^, indicating the fastest charge-transfer process and the most active electrode/electrolyte interface.

Overall, the impedance results demonstrate that Cu incorporation is an effective strategy to improve both interfacial charge transfer and Na^+^ diffusion in ZnO. The Zn_0.9_Cu_0.1_O electrode shows the most balanced kinetic behavior, including the lowest R_ct_, highest G_ct_, highest $D_{{Na}^{+}}$, highest J_0_, and highest K^0^. The slightly reduced performance of Zn_0.8_Cu_0.2_O compared with Zn_0.9_Cu_0.1_O suggests that excessive Cu content may disturb the defect structure and ion-transport pathway. Therefore, Zn_0.9_Cu_0.1_O is identified as the best-performing composition among the three compositions investigated in this study, rather than as the absolute optimized Cu concentration.

Spin-polarized density functional theory (DFT) calculations were performed to understand the electronic structure modification induced by Cu substitution in ZnO. The calculations were carried out using the Vienna Ab initio Simulation Package (VASP) [32,33]. The generalized gradient approximation with the Perdew–Burke–Ernzerhof exchange–correlation functional was employed [34,35]. The pristine wurtzite ZnO structure and Cu-substituted ZnO supercell were geometrically optimized before electronic structure analysis. A plane-wave cut-off energy of 500 eV was used, and Monkhorst–Pack k-point meshes of 6 × 6 × 7 and 4 × 4 × 7 were applied for pristine ZnO and Cu-substituted ZnO, respectively. The electronic convergence criterion was set to 10–5 eV, while the force convergence criterion was fixed at 1 meV Å^−1^. The defect formation energy (FE) is given by Equation (10):

(10) $$FE=E({Zn}_{32}{Cu}_{4}O_{36})-\frac{32\;E\left(Zn\right)+4E\left(Cu\right)+32E(O)}{n}$$

where E (Zn_32_Cu_4_O_36_) is the total E of the ZnO with 10% Cu substitution, E(Zn), E(Cu), and E(O) represent the total energy of isolated Zn, Cu and a 20× 20× 20 supercell of an O_2_ molecule, respectively. The calculated formation energies are −1.988 eV (Zn_0.9_Cu_0.1_O) and −1.996 eV (Zn_0.8_Cu_0.2_O), indicating the thermodynamic stability of both systems. The small variation (≈0.01 eV/atom) suggests Cu doping does not drastically alter stability but may influence electronic properties.

**Figure 9:**
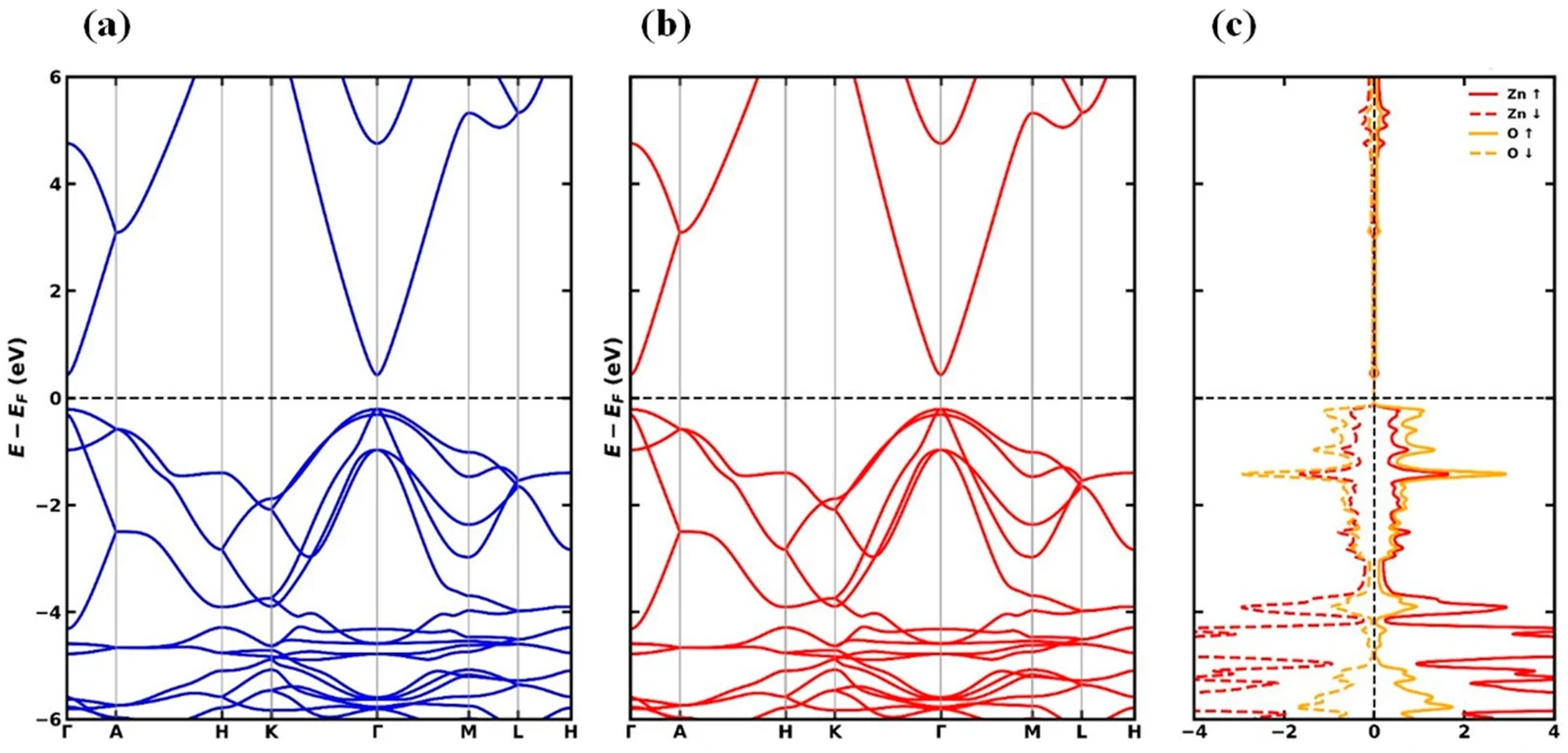
The calculated band structure of (**a**,**b**) ZnO for spin-up and spin-down channels, respectively. (**c**) represents the partial density of states (PDOS) of the pristine system.

**Figure 10:**
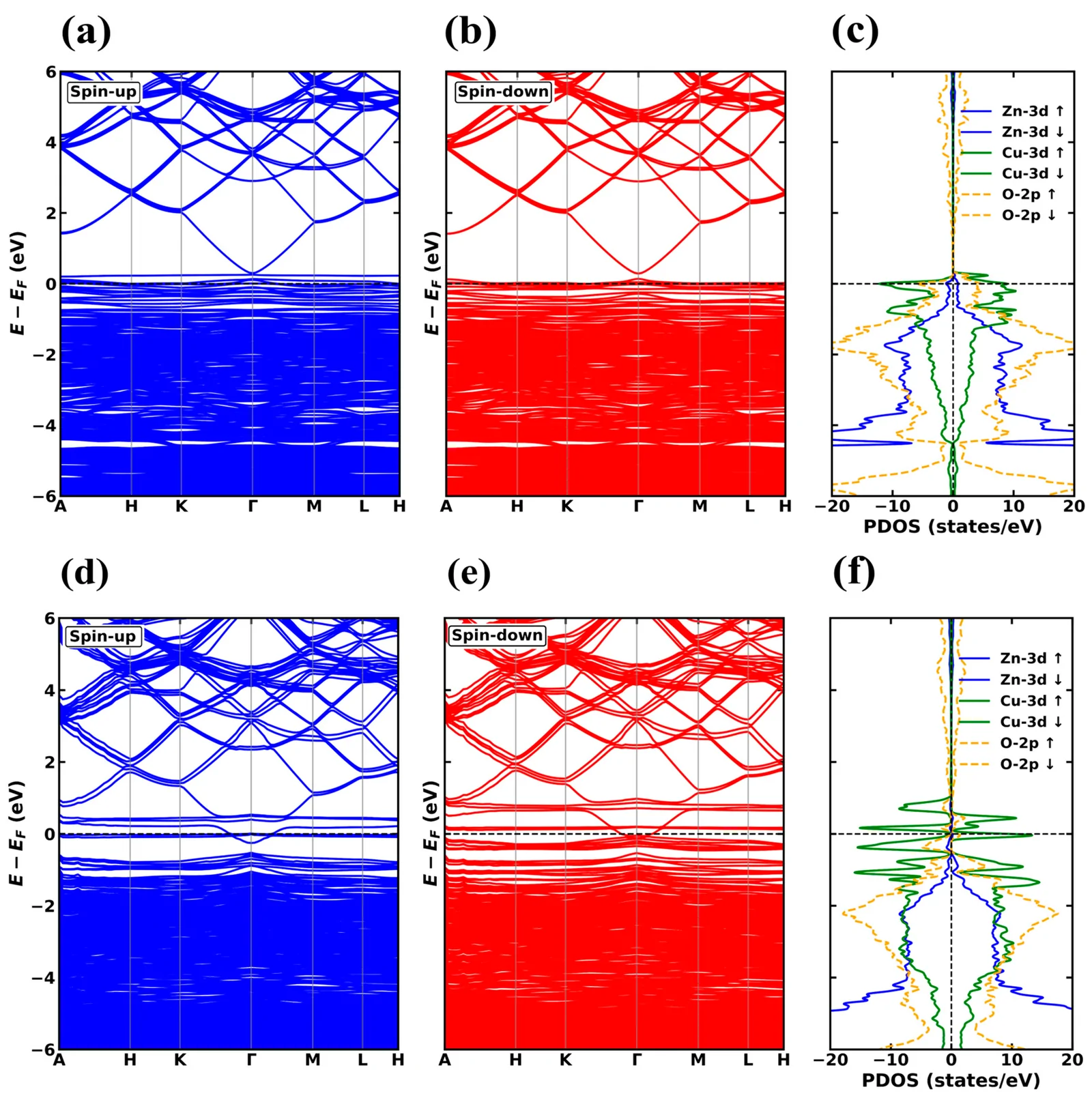
The calculated band structures of (**a**,**b**) ZnO with 10% Cu and (**d**,**e**) ZnO with 20% Cu substitution for spin-up and spin-down channels, respectively. (**c**,**f**) represent the partial density of states (PDOS) of pristine ZnO and ZnO with 10% and 20% Cu substitution systems, respectively.

The optimized structures confirm that the wurtzite ZnO framework is retained after Cu substitution. No severe structural collapse is observed in the optimized Cu-substituted model, indicating that Cu can be accommodated within the ZnO lattice under the selected substitutional configuration. This agrees with the experimental XRD results, where all diffraction peaks are indexed to the hexagonal wurtzite ZnO phase, and no secondary Cu-, CuO-, or Cu_2_O-related peaks are detected. Therefore, both the experimental and theoretical results suggest that Cu incorporation modifies the ZnO lattice without destroying the primary crystal framework. The spin-polarized band structures of pristine ZnO and Cu-substituted ZnO are shown in Figure 9a,b and Figure 10a,b,d,e.

The geometrical structures of bulk wurtzite ZnO and a 4 × 4 × 2 supercell of wurtzite ZnO with 10% Cu substitution were optimized, as presented in Figure 9. The optimized structures confirm that the wurtzite ZnO framework is retained after Cu substitution. No severe structural collapse is observed in the optimized Cu-substituted model, indicating that Cu can be accommodated within the ZnO lattice under the selected substitutional configuration. This agrees with the experimental XRD results, where all diffraction peaks are indexed to the hexagonal wurtzite ZnO phase, and no secondary Cu-, CuO-, or Cu_2_O-related peaks are detected. Therefore, both the experimental and theoretical results suggest that Cu incorporation modifies the ZnO lattice without destroying the primary crystal framework.

The spin-polarized band structures of pristine ZnO, Zn_0.9_Cu_0.1_O, and Zn_0.8_Cu_0.2_O are shown in Figure 9a,b, Figure 10a,b, and Figure 10d,e, respectively, while the corresponding partial density of states (PDOS) are presented in Figure 9c, Figure 10c, and Figure 10f, respectively. The Fermi level is set at 0 eV. For pristine ZnO, both spin-up and spin-down band structures exhibit semiconducting behavior, with a calculated band gap of approximately 0.64 eV. This value is lower than the experimental optical band gap obtained from DRS because the GGA-PBE functional is known to underestimate semiconductor band gaps. Therefore, the calculated band gap value should be used mainly to compare the electronic structure trend rather than as an absolute reproduction of the experimental optical band gap.

The PDOS of pristine ZnO shows that the valence-band region is mainly dominated by O-related states together with Zn contributions, while the conduction-band region is associated with Zn-derived states. The absence of significant electronic states at the Fermi level confirms the semiconducting character of pristine ZnO. After Cu substitution, the electronic band structure changes significantly. In contrast with pristine ZnO, the Cu-substituted ZnO system shows several electronic bands crossing or approaching the Fermi level in both spin channels for both systems. In contrast with pristine ZnO, the Cu-substituted ZnO system shows several electronic bands crossing or approaching the Fermi level in both spin channels. This indicates that Cu incorporation introduces additional electronic states near the Fermi level and strongly modifies the band-edge region of ZnO. The overlap of occupied and unoccupied states near (E_F_) suggests metallic or highly conductive behavior in the ideal Cu-substituted DFT model.

The PDOS analysis provides deeper insight into the origin of this electronic modification. For Cu-substituted ZnO, Cu-derived states appear near the Fermi level and strongly contribute to the electronic density around the band-edge region. These Cu-related states interact with O states, indicating Cu 3d–O2p orbital hybridization. This hybridization creates additional electronic states within or near the forbidden region of ZnO and reduces the effective energy separation between the valence and conduction bands. As a result, lower-energy electronic transitions become possible after Cu incorporation. Furthermore, the Cu-3d orbital reveals a dominant contribution near the Fermi level, as shown in the PDOS in Figure 10c,f. At x = 0.1, isolated Cu substitution generates localized Cu-3d mid-gap states that act as charge-trapping centers, narrowing the optical band gap and creating kinetic bottlenecks. In contrast, at x = 0.2, increased Cu–Cu spatial proximity induces strong Cu-3d orbital overlap, transforming these discrete mid-gap states into a continuous, delocalized metallic band near the Fermi level (E_F_). This electronic transition attenuates deep sub-band gap optical transitions to yield the apparent optical gap recovery while substantially enhancing bulk electronic conductivity, thereby accelerating reaction kinetics and fully explaining the experimentally observed non-monotonic performance.

The DFT result is consistent with the DRS analysis. Experimentally, pristine ZnO shows a direct optical band gap of 3.08 eV, while Zn_0.9_Cu_0.1_O exhibits a significantly reduced band gap of 2.66 eV. The strong band gap narrowing observed in DRS can be explained by the Cu-induced electronic states predicted by DFT. These states act as intermediate or band-tail-like levels near the band edges, decreasing the energy required for electronic excitation. The electronic structure modification is also important for sodium-ion battery performance. ZnO-based conversion/alloying anodes often suffer from sluggish charge transfer because of limited intrinsic electronic conductivity. When Cu states are introduced near the Fermi level, the availability of electronic states for charge transport increases. This can improve electronic movement through the active material and reduce the resistance associated with interfacial charge transfer. Therefore, Cu substitution can make the ZnO electrode electronically more accessible during sodiation/desodiation. Moreover, the improved electronic accessibility suggested by DFT agrees with the EIS results. Among the investigated samples, Zn_0.9_Cu_0.1_O exhibits the lowest specific charge-transfer resistance and the highest charge-transfer conductance. This means that the Cu-induced electronic states predicted by DFT are not only important for optical band gap narrowing but also help explain the faster electrochemical reaction kinetics. A lower charge-transfer resistance allows electrons to move more efficiently during the conversion/alloying reaction of ZnO, leading to improved rate capability and cycling performance. It is important to note that the metallic behavior predicted for the Cu-substituted model represents an ideal periodic substitutional system. The experimental sample may still show semiconducting behavior because real materials contain finite particle size, grain boundaries, local disorder, and non-uniform dopant environments. Therefore, the DFT result should not be interpreted as proof that the experimental Zn_0.9_Cu_0.1_O sample is fully metallic. Instead, it should be understood as evidence that Cu substitution introduces electronic states near the Fermi level and strongly enhances the electronic conductivity tendency of ZnO.

## 4. Conclusions

In this work, composition-tuned Zn_1−x_Cu_x_O nanoparticles were successfully synthesized using a simple co-precipitation method and investigated as wurtzite oxide anodes for sodium-ion batteries. The main objective was not only to improve the sodium storage behavior of ZnO but also to establish a meaningful electro-optical correlation between band-edge modulation and charge-transfer kinetics. XRD analysis confirmed that all samples retained the hexagonal wurtzite ZnO structure without detectable secondary phases, indicating that composition tuning did not destroy the main crystal framework. Morphological analysis showed that the Zn_0.9_Cu_0.1_O sample exhibited a more refined and uniform particle distribution compared with pristine ZnO. DRS analysis further revealed a clear composition-dependent optical response. The direct band gap decreased from 3.08 eV for pristine ZnO to 2.66 eV for Zn_0.9_Cu_0.1_O, indicating strong band-edge modification and improved electronic accessibility. However, the band gap partially recovered to 2.99 eV for Zn_0.8_Cu_0.2_O, showing that the electronic improvement was not proportional to increasing Cu content. DFT calculations supported the experimental DRS results by showing that Cu incorporation introduces additional electronic states near the Fermi level. These electronic states can reduce the electronic barrier for charge movement and provide a theoretical explanation for the improved charge-transfer behavior. The electrochemical results confirmed that the optical and electronic trends were closely related to sodium storage kinetics. Zn_0.9_Cu_0.1_O delivered the lowest specific charge-transfer resistance of 185.15 Ω cm^2^, the highest charge-transfer conductance of 8.32 mS, and the highest Na^+^ diffusion coefficient of 5.77 × 10^−11^ cm^2^ s^−1^. In contrast, pristine ZnO showed higher specific charge-transfer resistance and lower diffusion kinetics, while Zn_0.8_Cu_0.2_O showed intermediate behavior. This confirms that the strongest band gap narrowing and most favorable electronic-state modification correspond to the best interfacial charge-transfer kinetics. Therefore, the superior performance of Zn_0.9_Cu_0.1_O originates from a balanced combination of particle-size uniformity, optical band-edge modulation, and improved electrochemical charge-transfer kinetics. The results show that DRS, DFT, and EIS should not be treated as separate analyses; instead, they can be connected to explain the sodium storage behavior of semiconductor-type oxide anodes. This study demonstrates that optical band-edge regulation can be used as a practical descriptor for evaluating the charge-transfer kinetics in composition-tuned wurtzite oxide anodes for sodium-ion storage. A comparison of the electrochemical performance of the present ZnO-based electrodes with previously reported ZnO anodes for SIBs is summarized in Table 2 [7,8,15,17,18,19,22,36,37].

## Figures and Tables

**Table 1:** XRD analysis of ZnO and Cu-doped ZnO at different nanoparticle concentrations, including unit cell volume (V), lattice constants (a=b, c), dislocation density, and crystallite size (D).

Parameter	ZnO	Zn_0.9_Cu_0.1_O	Zn_0.8_Cu_0.2_O
Average size D (nm)	29.91	25.66	28.72
d_101_ (Å)	2.4479	2.4574	2.4616
a = b (Å)	3.253	3.216	3.227
c (Å)	5.199	5.116	5.168
V(Å)^3^	47.53	45.76	46.55
Macrostrain (10^−3^)	1.62	7.50	5.91
Dislocation density δ/10^14^ m^−2^	8.27	15.19	12.12

**Table 2:** Specific capacity and cycle performance comparison of ZnO anodes used for SIBs.

Composition/Morphology	Synthesis Approach	Initial Discharge Capacity (mAh g^−1^)	Specific Capacity (Cycles) (mAh g^−1^)	Current Density (A g^−1^)	Rate Performance (mAh g^−1^)	Current Density (A g^−1^)	Ref.
Zn_0.8_Mn_0.2_O/Uniform	Co-precipitation method	482.76	322.2 (100th)	0.1	239.9	5.000	[22]
ZnO-rGO/Nano rods	Electrochemical process	376.4/-	188.6 (150th)	0.1	98.7	1.600	[15]
ZnO-ZnS@NC/rod-like	Facile multi-step strategy	1009.8	431.6 (10th)	0.1	123	1	[8]
ZnO@NF composites	Atomic layer deposition	197.7	65.1 (400)	0.03	30.9	1	[7]
ZnO-carbon/core-shell	Hydrothermal method	389.4	100 (200th)	0.1	70	0.3	[19]
Mn-doped Sn-ZnO/rod-shaped	Co-precipitation	-	395.1 (300)	0.1	332	5.00	[17]
ZnO-rGO-C/hexahedron	Solvothermal synthesis	1579.6	300 (100th)	0.032	162	0.2 C	[18]
NiO/ZnO	Hydrothermal method	861.2/331.9	300 (150th)	0.05	154	2.500	[36]
NG-ZnO/NiO	Solvothermal process	-	235 (400th)	0.018	118	1.860	[37]
Zn_0.9_Cu_0.1_O	Co-precipitation method	411.42	292.17 (200th)	0.1	219.97	5.0	This Work

## Data Availability

The original contributions presented in this study are included in the article. Further inquiries can be directed to the corresponding authors.
